# Repeatability of Brain Volume Measurements Made with the Atlas-based Method from T_1_-weighted Images Acquired Using a 0.4 Tesla Low Field MR Scanner

**DOI:** 10.2463/mrms.mp.2015-0107

**Published:** 2016-02-03

**Authors:** Masami GOTO, Makoto SUZUKI, Shinya MIZUKAMI, Osamu ABE, Shigeki AOKI, Tosiaki MIYATI, Michinari FUKUDA, Tsutomu GOMI, Tohoru TAKEDA

**Affiliations:** 1 School of Allied Health Sciences, Kitasato University, 1-15-1 Minami-ku, Sagamihara, Kanagawa 252-0373, Japan; 2 Graduate School of Medical Sciences, Kitasato University; 3 Department of Radiology, Nihon University School of Medicine; 4 Department of Radiology, Juntendo University; 5 Graduate School of Medical Science, Kanazawa University

**Keywords:** atlas-based method, brain volumetry, low static magnetic field, repeatability, WFU PickAtlas

## Abstract

**Purpose:**

An understanding of the repeatability of measured results is important for both the atlas-based and voxel-based morphometry (VBM) methods of magnetic resonance (MR) brain volumetry. However, many recent studies that have investigated the repeatability of brain volume measurements have been performed using static magnetic fields of 1–4 tesla, and no study has used a low-strength static magnetic field. The aim of this study was to investigate the repeatability of measured volumes using the atlas-based method and a low-strength static magnetic field (0.4 tesla).

**Materials and Methods:**

Ten healthy volunteers participated in this study. Using a 0.4 tesla magnetic resonance imaging (MRI) scanner and a quadrature head coil, three-dimensional T_1_-weighted images (3D-T_1_WIs) were obtained from each subject, twice on the same day. VBM8 software was used to construct segmented normalized images [gray matter (GM), white matter (WM), and cerebrospinal fluid (CSF) images]. The regions-of-interest (ROIs) of GM, WM, CSF, hippocampus (HC), orbital gyrus (OG), and cerebellum posterior lobe (CPL) were generated using WFU PickAtlas. The percentage change was defined as

[100 × (measured volume with first segmented image−mean volume in each subject)/(mean volume in each subject)]

The average percentage change was calculated as the percentage change in the 6 ROIs of the 10 subjects.

**Results:**

The mean of the average percentage changes for each ROI was as follows: GM, 0.556%; WM, 0.324%; CSF, 0.573%; HC, 0.645%; OG, 1.74%; and CPL, 0.471%. The average percentage change was higher for the orbital gyrus than for the other ROIs.

**Conclusion:**

We consider that repeatability of the atlas-based method is similar between 0.4 and 1.5 tesla MR scanners. To our knowledge, this is the first report to show that the level of repeatability with a 0.4 tesla MR scanner is adequate for the estimation of brain volume change by the atlas-based method.

## Introduction

Magnetic resonance (MR) brain volumetry using T_1_-weighted images (T_1_WIs) is generally conducted with either the voxel-based morphometry method^1^ or the region-of-interest (ROI) method. The ROI method is further subdivided into the manually traced ROI^2–4^ and atlas-based^5,6^ methods. The manual measurement method is difficult, time-consuming, and susceptible to rater bias, while the atlas-based method employs semi-automated algorithms and is operator-independent.

Because the repeatability of measured results is important in both methods, several recent studies have investigated the repeatability of brain volume measurements.^7–13^

A search for studies on repeatability for brain volume evaluation returned 21 review articles^14–34^ and 7 original research articles^7–13^; however, the static magnetic field in these studies varied from 1 to 4 tesla and we found none that used a low-strength static magnetic field. Therefore, the aim of this study was to investigate the repeatability of measured volumes using the atlas-based method with a low-strength static magnetic field (0.4 tesla).

## Materials and Methods

### Subjects

Ten healthy volunteers participated in the study (4 males, 6 females; mean age, 34.4 ± 9.9 years; age range, 22–47 years). Using a 0.4 tesla MR scanner, three-dimensional T_1_WI (3D-T_1_WI) was obtained from each subject, twice serially on the same day, and T_2_WI was obtained from each subject for observation of white matter (WM) lesions. The 3D-T_1_WI and T_2_WI were inspected by a board-certified radiologist, who found none of the following findings in any subject: brain tumor, infarction, hemorrhage, brain atrophy, or WM lesions graded higher than grade 2 of Fazekas’s classification.^35^ The protocol was approved by the ethical committee of our institution. After the study had been explained to each subject, written informed consent was obtained from all participants.

### MRI scanning protocol

Using a 0.4 tesla scanner (Aperto Lucent, Hitachi) and quadrature head coil, we employed a 3D gradient echo with inversion recovery (3D-GEIR) sequence to obtain 100 contiguous sagittal T_1_-weighted images with a slice thickness of 2.0 mm (reconstruction pitch 1.0 mm), repetition time/echo time = 5.9/2.5 ms, inversion time = 600 ms, flip angle = 12°, field of view = 24 cm, number of excitations = 1, and 256 × 256 pixel matrix.

### Image preprocessing and statistical analyses for the atlas-based method

We changed only one parameter from the default setting of the VBM8 tool implemented in Statistical Parametric Mapping 8 (SPM8) software (Wellcome Department of Imaging Neuroscience Group, London, UK; http://www.fil.ion.ucl.ac.uk/spm): the affine regularization space template from the International Consortium for Brain Mapping was changed from “European brain” to “East Asian brain,” as all the subjects in our study were Japanese. The 3D-T_1_WI of the 10 subjects were then processed using VBM8, and the resulting segmented gray matter (GM), WM, and cerebrospinal fluid (CSF) images were normalized into Montreal Neurological Institute (MNI) space.

ROIs were obtained by WFU PickAtlas (Talairach brain atlas theory).^5^ To enable comparison of the present results with those of a previous report^13^ that investigated repeatability with a 1.5 tesla scanner, we used the same ROIs as in that study: GM, WM, CSF, hippocampus (HC), orbital gyrus (OG), and cerebellum posterior lobe (CPL). Volume measurements for GM, HC, OG, and CPL were performed using segmented GM images, while those for WM and CSF were performed using segmented WM or CSF images. The content rate of the tissue within each ROI was measured in all segmented images. The percentage change was defined as:

(1) $$\text{The}\;\text{percentage}\;\text{change}=100\times \frac{\text{measured}\;\text{volume}\;\text{with}\;\text{first}\;\text{segmented}\;\text{image}-\text{mean}\;\text{volume}\;\text{in}\;\text{each}\;\text{subject}}{\text{mean}\;\text{volume}\;\text{in}\;\text{each}\;\text{subject}}$$

The average percentage change was calculated as the percentage changes for GM, WM, CSF, HC, OG, and CPL for the 10 subjects. That is, a low average percentage change denoted high repeatability. The statistical significance of differences between the different ROIs was examined using analysis of variance (ANOVA), and the Tukey–Kramer method was used as a post hoc test with SAS-JMP software (SAS Institute, Cary, North Carolina, USA). The level of statistical significance in both tests was set as *P* < 0.05.

### Image preprocessing and statistical analyses for the VBM method

We also investigated the repeatability of measured volumes with a low-strength static magnetic field (0.4 tesla) using the VBM method, employing the segmented images obtained as described in the previous section (Image preprocessing and statistical analyses for atlas-based method). These segmented images were smoothed with a Gaussian kernel of 8 mm full width at half maximum, which is the default setting of SPM8. For each subject, we defined the mean images as:

(2) $$\text{The}\;\text{mean}\;\text{image}=\frac{\text{smoothed}\;\text{segmented}\;\text{images}\;\text{of}\;\text{the}\;\text{first}\;3\text{D}-{\text{T}}_{1}\text{WI}+\text{smoothed}\;\text{segmented}\;\text{images}\;\text{of}\;\text{the}\;\text{second}\;3\text{D}-{\text{T}}_{1}\text{WI}}{2}$$

And, we defined the percentage change images as:

(3) $$\text{The}\;\text{percentage}\;\text{change}\;\text{image}=100\times \frac{\text{smoothed}\;\text{segmented}\;\text{images}\;\text{of}\;\text{the}\;\text{first}\;3\text{D}-{\text{T}}_{1}\text{WI}-\text{mean}\;\text{images}}{\text{mean}\;\text{images}}$$

We then made repeatability maps (a mean map of the percentage change images of the 10 subjects) for each tissue type (GM, WM, and CSF).

## Results

### Repeatability using the atlas-based method

**Fig. 1.**
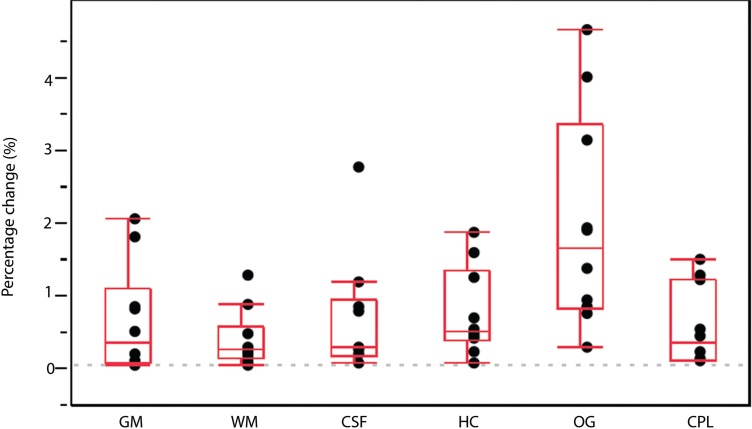
Repeatability with the atlas-based method. The percentage changes in each ROI are shown with the box plot. The box plot shows median, maximum, and minimum value below inner fence, first and third quartile, and outliers. Analysis of variance revealed a significant difference in average percentage change for all six regions (*P* < 0.05). In addition, the Tukey–Kramer method revealed significant difference (*P* < 0.05) for OG vs. GM, vs. WM, vs. CSF, vs. HC, and vs. CPL. The percentage change value was larger for OG than for all the other ROIs. CPL, cerebellum posterior lobe; CSF, cerebrospinal fluid; GM, gray matter; HC, hippocampus; OG, orbital gyrus; ROI, region-of-interest; WM, white matter.

The average percentage changes using the atlas-based method are shown in Fig. 1. The average percentage changes on each ROI were as follows: GM, 0.556 ± 0.657% (mean value ± standard deviation); WM, 0.324 ± 0.355%; CSF, 0.573 ± 0.742%; HC, 0.645 ± 0.541%; OG, 1.74 ± 1.33%; and CPL, 0.471 ± 0.494%. ANOVA revealed a significant difference in average percentage change for all six regions (*P* < 0.05). The average percentage change was highest for OG, which indicates that repeatability was relatively low for OG.

### Repeatability using the VBM method

**Fig. 2.**
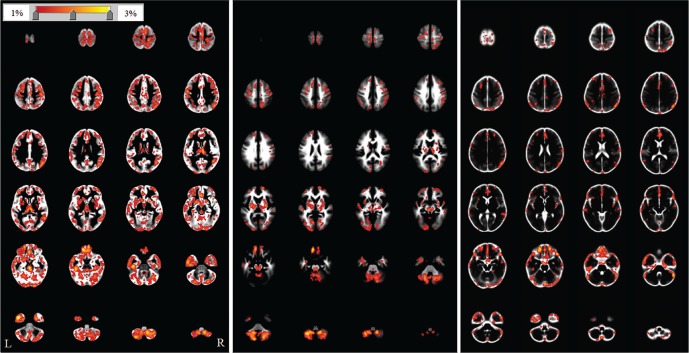
The repeatability map for the voxel-based morphometry method. The repeatability maps are superimposed on the template image for each tissue (gray matter, white matter, and cerebrospinal fluid) processed with SPM8. The color bar (top left) indicates the percentage change. R and L are the right and left sides of the subjects, respectively. High-value areas (maximum values) were found near the skull base in the gray matter (3.50%), white matter (3.16%), and cerebrospinal fluid (3.02%) images.

Repeatability maps are shown in Fig. 2. In the repeatability map, superior repeatability is indicated as a low value of the percentage changes in a voxel. High-value areas (maximum values) were found near the skull base in the GM (3.50%), WM (3.16%), and CSF (3.02%) images.

## Discussion

We have documented the average percentage changes in GM, WM, CSF, HC, OG, and CPL using the atlas-based method and employing a 0.4 tesla MR scanner. The means of the average percentage change for each ROI were as follows: GM, 0.556%; WM, 0.324%; CSF, 0.573%; HC, 0.645%; OG, 1.74%; and CPL, 0.471%. In a previous report^13^ that used the atlas-based method and a 1.5 tesla MR scanner, the means of the average percentage change for each ROI were as follows: GM, 0.482%; WM, 0.375%; CSF, 0.731%; HC, 0.864%; OG, 1.69%; and CPL, 0.854%. Other previous reports showed coefficients of variation (100 × standard deviation of the differences/overall mean) of 0.41%, 0.59%, and 1.07% for GM, WM, and CSF, respectively, scanning 10 subjects twice on the same day with a 2 tesla scanner.^36^ We cannot compare significant differences between the results of the present study and the previous report^13^ because of differences in study design. However, we consider that the atlas-based method with a 0.4 tesla MR scanner has similar repeatability to that with a 1.5 tesla MR scanner. The present results show that the level of repeatability in the atlas-based method with a 0.4 tesla MR scanner is adequate for the estimation of brain volume change because the repeatability is similar to that obtained with a 1.5 tesla MR scanner. A 1.5 tesla MR scanner is the most common field strength used in previous studies that have estimated brain volume change.^14–34^

Image distortion is more severe for a high-strength than a low-strength static magnetic field. The position of the OG near the skull base makes it particularly affected by the magnetic susceptibility of the nasal sinuses, and we therefore expected superior repeatability with 0.4 tesla than with 1.5 tesla for OG. However, the present results show low repeatability for OG with 0.4 tesla compared with other ROIs, similar to the results of a previous report^13^ with 1.5 tesla. Based on the similarity of the results between 0.4 and 1.5 tesla, we propose that the main cause of low repeatability for OG is misregistration in the spatial normalizing step with VBM8, rather than the magnetic susceptibility of the nasal sinuses.

Fig. 1 showed repeatability (average percentage changes) using the atlas-based method, but variety inside ROI was not shown. And we used major ROI for the investigation of repeatability, but other regions also are used by morphometric researchers. We investigated repeatability with voxel-based method because we think that investigation for local area in whole brain is needed for morphometric researchers. The repeatability maps in Fig. 2 show that the highest values are found near the skull base in the GM, WM, and CSF images. These results are consistent with those in the section “Repeatability by the atlas-based method.” OG shows a high-value area on the repeatability map with the atlas-based method. GM, WM, and CSF with the atlas-based method include high-value areas on the repeatability map, but also include low-value areas. Therefore, the mean within the ROI did not show high values for these regions, compared with OG.

Commonly, a scanner with low-strength static magnetic field have low signal-to-noise ratio (SNR) compared with a high-strength scanner. The previous report^7^ for the association between brain tissue volumes and effects of changes in SNR showed that SNR in 3D-T_1_WI with 1.5 tesla scanner for VBM is 28.6 ± 2.5. We performed additional measurement for SNR in our images with a method like the previous report. SNR was defined as the mean voxel intensity in the right cerebral subcortical WM ROI divided by the standard deviation of the intensity distribution. As a result, SNR in our images was 26.2 ± 2.9. We obtained 3D-T_1_WI with the scanning protocol that imaging time is about 10 minutes. We employed scanning protocol with longer scan time compared with 1.5 tesla scanner because SNR is an important factor for repeatability in VBM. Longer scan time increases SNR but incidence of artifact from head motion also may increase for extended scan time. Therefore, we think that 2-mm thickness is optimal, because thinner slice leads to lower SNR and thicker slice leads to lower spatial resolution.

The major limitation of the present study is its small sample size. However, we consider that the results of a large sample may show similar trends because the present range of percentage changes with the atlas-based method is similar to that reported previously.^13^ The second limitation is that we cannot compare accuracy between 0.4 and 1.5 tesla field strengths because there is no gold standard for brain volumetry. The third limitation is that we cannot show the result for subject with cortex atrophy. To confirm whether 0.4 tesla scanners are suitable for brain volumetry, it would be necessary to compare detectability between 0.4 and 1.5 tesla in a future study.

## Conclusion

To our knowledge, this is the first report to show that the level of repeatability obtained with the atlas-based method using a 0.4 tesla MR scanner is adequate for estimation of brain volume change.

## References

[1] Ashburner J Friston KJ Voxel-based morphometry—the methods. Neuroimage 2000; 11 ( 6 Pt 1): 805– 821. 10860804 10.1006/nimg.2000.0582

[2] Courchesne E Chisum HJ Townsend J Normal brain development and aging: quantitative analysis at in vivo MR imaging in healthy volunteers. Radiology 2000; 216: 672– 682. 10966694 10.1148/radiology.216.3.r00au37672

[3] Pfefferbaum A Mathalon DH Sullivan EV Rawles JM Zipursky RB Lim KO A quantitative magnetic resonance imaging study of changes in brain morphology from infancy to late adulthood. Arch Neurol 1994; 51: 874– 887. 8080387 10.1001/archneur.1994.00540210046012

[4] Luft AR Skalej M Schulz JB Patterns of age-related shrinkage in cerebellum and brainstem observed in vivo using three-dimensional MRI volumetry. Cereb Cortex 1999; 9: 712– 721. 10554994 10.1093/cercor/9.7.712

[5] Maldjian JA Laurienti PJ Kraft RA Burdette JH An automated method for neuroanatomic and cytoarchitectonic atlas-based interrogation of fMRI data sets. Neuroimage 2003; 19: 1233– 1239. 12880848 10.1016/s1053-8119(03)00169-1

[6] Gonoi W Abe O Yamasue H Age-related changes in regional brain volume evaluated by atlas-based method. Neuroradiology 2010; 52: 865– 873. 20033142 10.1007/s00234-009-0641-5

[7] Shuter B Yeh IB Graham S Au C Wang SC Reproducibility of brain tissue volumes in longitudinal studies: effects of changes in signal-to-noise ratio and scanner software. Neuroimage 2008; 41: 371– 379. 18394925 10.1016/j.neuroimage.2008.02.003

[8] Ewers M Teipel SJ Dietrich O Multicenter assessment of reliability of cranial MRI. Neurobiol Aging 2006; 27: 1051– 1059. 16169126 10.1016/j.neurobiolaging.2005.05.032

[9] Jovicich J Czanner S Greve D Reliability in multi-site structural MRI studies: effects of gradient non-linearity correction on phantom and human data. Neuroimage 2006; 30: 436– 443. 16300968 10.1016/j.neuroimage.2005.09.046

[10] Jovicich J Czanner S Han X MRI-derived measurements of human subcortical, ventricular and intracranial brain volumes: reliability effects of scan sessions, acquisition sequences, data analyses, scanner upgrade, scanner vendors and field strengths. Neuroimage 2009; 46: 177– 192. 19233293 10.1016/j.neuroimage.2009.02.010 PMC2866077

[11] Leow AD Klunder AD Jack CR Jr Longitudinal stability of MRI for mapping brain change using tensor-based morphometry. Neuroimage 2006; 31: 627– 640. 16480900 10.1016/j.neuroimage.2005.12.013 PMC1941663

[12] Han X Jovicich J Salat D Reliability of MRI-derived measurements of human cerebral cortical thickness: the effects of field strength, scanner upgrade and manufacturer. Neuroimage 2006; 32: 180– 194. 16651008 10.1016/j.neuroimage.2006.02.051

[13] Goto M Miyati T Abe O Repeatability of measured brain volume by atlas-based method using T1-weighted image. J Digit Imaging 2012; 25: 173– 178. 21773867 10.1007/s10278-011-9412-z PMC3264725

[14] Celle S Delon-Martin C Roche F Barthélémy JC Pepin JL Dojat M Desperately seeking grey matter volume changes in sleep apnea: A methodological review of magnetic resonance brain voxel-based morphometry studies. Sleep Med Rev 2015 . [Epub ahead of print] 10.1016/j.smrv.2015.03.001 26140868

[15] Li L Wu M Liao Y Grey matter reduction associated with posttraumatic stress disorder and traumatic stress. Neurosci Biobehav Rev 2014; 43: 163– 172. 24769403 10.1016/j.neubiorev.2014.04.003

[16] Shao N Yang J Li J Shang HF Voxelwise meta-analysis of gray matter anomalies in progressive supranuclear palsy and Parkinson’s disease using anatomic likelihood estimation. Front Hum Neurosci 2014; 8: 63. 24600372

[17] Cao B Tang Y Li J Zhang X Shang HF Zhou D A meta-analysis of voxel-based morphometry studies on gray matter volume alteration in juvenile myoclonic epilepsy. Epilepsy Res 2013; 106: 370– 377. 23962795 10.1016/j.eplepsyres.2013.07.003

[18] Yang J Shao N Li J Shang H Voxelwise meta-analysis of white matter abnormalities in progressive supranuclear palsy. Neurol Sci 2014; 35: 7– 14. 23912687 10.1007/s10072-013-1512-8

[19] Shi HC Zhong JG Pan PL Gray matter atrophy in progressive supranuclear palsy: meta-analysis of voxel-based morphometry studies. Neurol Sci 2013; 34: 1049– 1055. 23543378 10.1007/s10072-013-1406-9

[20] Ahmed F Ras J Seedat S Volumetric structural magnetic resonance imaging findings in pediatric posttraumatic stress disorder and obsessive compulsive disorder: a systematic review. Front Psychol 2012; 3: 568. 23272001 10.3389/fpsyg.2012.00568 PMC3530132

[21] Pan P Shi H Zhong J Chronic smoking and brain gray matter changes: evidence from meta-analysis of voxel-based morphometry studies. Neurol Sci 2013; 34: 813– 817. 23207549 10.1007/s10072-012-1256-x

[22] Pan PL Shi HC Zhong JG Gray matter atrophy in Parkinson’s disease with dementia: evidence from meta-analysis of voxel-based morphometry studies. Neurol Sci 2013; 34: 613– 619. 23184330 10.1007/s10072-012-1250-3

[23] Pan PL Song W Yang J Gray matter atrophy in behavioral variant frontotemporal dementia: a meta-analysis of voxel-based morphometry studies. Dement Geriatr Cogn Disord 2012; 33: 141– 148. 22722668 10.1159/000338176

[24] Selvaraj S Arnone D Job D Grey matter differences in bipolar disorder: a meta-analysis of voxel-based morphometry studies. Bipolar Disord 2012; 14: 135– 145. 22420589 10.1111/j.1399-5618.2012.01000.x

[25] Yang J Pan P Song W Voxelwise meta-analysis of gray matter anomalies in Alzheimer’s disease and mild cognitive impairment using anatomic likelihood estimation. J Neurol Sci 2012; 316: 21– 29. 22385679 10.1016/j.jns.2012.02.010

[26] Modinos G Costafreda SG van Tol MJ McGuire PK Aleman A Allen P Neuroanatomy of auditory verbal hallucinations in schizophrenia: a quantitative meta-analysis of voxel-based morphometry studies. Cortex 2013; 49: 1046– 1055. 22370252 10.1016/j.cortex.2012.01.009

[27] Li J Pan P Huang R Shang H A meta-analysis of voxel-based morphometry studies of white matter volume alterations in Alzheimer’s disease. Neurosci Biobehav Rev 2012; 36: 757– 763. 22192882 10.1016/j.neubiorev.2011.12.001

[28] Rizzo G Manners D Vetrugno R Combined brain voxel-based morphometry and diffusion tensor imaging study in idiopathic restless legs syndrome patients. Eur J Neurol 2012; 19: 1045– 1049. 22175823 10.1111/j.1468-1331.2011.03604.x

[29] Li J Zhang Z Shang H A meta-analysis of voxel-based morphometry studies on unilateral refractory temporal lobe epilepsy. Epilepsy Res 2012; 98: 97– 103. 22024190 10.1016/j.eplepsyres.2011.10.002

[30] Pan PL Song W Shang HF Voxel-wise meta-analysis of gray matter abnormalities in idiopathic Parkinson’s disease. Eur J Neurol 2012; 19: 199– 206. 21762435 10.1111/j.1468-1331.2011.03474.x

[31] Fusar-Poli P Borgwardt S Crescini A Neuroanatomy of vulnerability to psychosis: a voxel-based meta-analysis. Neurosci Biobehav Rev 2011; 35: 1175– 1185. 21168439 10.1016/j.neubiorev.2010.12.005

[32] Bora E Fornito A Yücel M Pantelis C Voxelwise meta-analysis of gray matter abnormalities in bipolar disorder. Biol Psychiatry 2010; 67: 1097– 1105. 20303066 10.1016/j.biopsych.2010.01.020

[33] Keller SS Roberts N Voxel-based morphometry of temporal lobe epilepsy: an introduction and review of the literature. Epilepsia 2008; 49: 741– 757. 18177358 10.1111/j.1528-1167.2007.01485.x

[34] Honea R Crow TJ Passingham D Mackay CE Regional deficits in brain volume in schizophrenia: a meta-analysis of voxel-based morphometry studies. Am J Psychiatry 2005; 162: 2233– 2245. 16330585 10.1176/appi.ajp.162.12.2233

[35] Fazekas F Chawluk JB Alavi A Hurtig HI Zimmerman RA MR signal abnormalities at 1.5 T in Alzheimer’s dementia and normal aging. AJR Am J Roentgenol 1987; 149: 351– 356. 3496763 10.2214/ajr.149.2.351

[36] Good CD Johnsrude IS Ashburner J Henson RN Friston KJ Frackowiak RS A voxel-based morphometric study of ageing in 465 normal adult human brains. Neuroimage 2001; 14: 21– 36. 11525331 10.1006/nimg.2001.0786

